# Flexible Sensors Array Based on Frosted Microstructured Ecoflex Film and TPU Nanofibers for Epidermal Pulse Wave Monitoring

**DOI:** 10.3390/s23073717

**Published:** 2023-04-03

**Authors:** Xue Wang, Zhiping Feng, Gaoqiang Zhang, Luna Wang, Liang Chen, Jin Yang, Zhonglin Wang

**Affiliations:** 1 State Key Laboratory of Coal Mine Disaster Dynamics and Control, Chongqing University, Chongqing 400044, China; 2 Key Laboratory of Optoelectronic Technology and Systems Ministry of Education, Department of Optoelectronic Engineering, Chongqing University, Chongqing 400044, China; 3 Beijing Institute of Nanoenergy and Nanosystems, Chinese Academy of Sciences, Beijing 100083, China

**Keywords:** health monitoring, pulse wave monitoring, pulse wave velocity, pressure sensors array

## Abstract

Recent advances in flexible pressure sensors have fueled increasing attention as promising technologies with which to realize human epidermal pulse wave monitoring for the early diagnosis and prevention of cardiovascular diseases. However, strict requirements of a single sensor on the arterial position make it difficult to meet the practical application scenarios. Herein, based on three single-electrode sensors with small area, a 3 × 1 flexible pressure sensor array was developed to enable measurement of epidermal pulse waves at different local positions of radial artery. The designed single sensor holds an area of 6 × 6 mm^2^, which mainly consists of frosted microstructured Ecoflex film and thermoplastic polyurethane (TPU) nanofibers. The Ecoflex film was formed by spinning Ecoflex solution onto a sandpaper surface. Micropatterned TPU nanofibers were prepared on a fluorinated ethylene propylene (FEP) film surface using the electrospinning method. The combination of frosted microstructure and nanofibers provides an increase in the contact separation of the tribopair, which is of great benefit for improving sensor performance. Due to this structure design, the single small-area sensor was characterized by pressure sensitivity of 0.14 V/kPa, a response time of 22 ms, a wide frequency band ranging from 1 to 23 Hz, and stability up to 7000 cycles. Given this output performance, the fabricated sensor can detect subtle physiological signals (e.g., respiration, ballistocardiogram, and heartbeat) and body movement. More importantly, the sensor can be utilized in capturing human epidermal pulse waves with rich details, and the consistency of each cycle in the same measurement is as high as 0.9987. The 3 × 1 flexible sensor array is employed to acquire pulse waves at different local positions of the radial artery. In addition, the time domain parameters including pulse wave transmission time (PTT) and pulse wave velocity (PWV) can be obtained successfully, which holds promising potential in pulse-based cardiovascular system status monitoring.

## 1. Introduction

Pulse wave velocity (PWV) is one of the important indicators of cardiovascular risk that can comprehensively reflect the damage of various risk factors to blood vessels and is an independent predictor of cardiovascular events [1,2]. The devices used for PWV detection in clinical settings have the characteristics of large volume and inconvenient operation, which limits the application scenarios of clinical devices in future personalized autonomous health monitoring. With the vigorous development of flexible sensing technology in the past few years, numerous flexible sensors have been developed to monitor various signals related to people, including body movements [3,4], postures [5], and other human physiological signals (e.g., respiration [6,7,8], ballistocardiogram [9], epidermal pulse wave [10,11]). As one of the most important physiological signals, pulse wave parameters (e.g., strength, shape, rhythm, duration, width, and transition time) lend insights into individuals’ health [12]. Pulse wave parameters are often considered an important information source for diagnosing health status and the cause of diseases in traditional Chinese medicine (TCM) [13,14]. The successful application of flexible sensing technology based on different principles (including piezoresistivity [15,16,17,18,19,20,21,22], capacitance [23,24,25,26,27,28,29,30], piezoelectricity [31,32,33,34], and triboelectric [35,36,37,38,39,40]) in pulse signal monitoring provides a new convenient and noninvasive detection form for PWV monitoring. Based on different material and structural design, flexible pressure sensors based on different principles demonstrate the excellent capability of pulse wave monitoring. As shown in Table S1, introducing different microstructures into the sensor structure can give the flexible pressure sensor good sensing ability, allow it to have its own suitable application scenarios.

Current work based on flexible sensing technology mostly focuses on the study of a single sensor. In the process of PWV monitoring, two independent sensors are often used to simultaneously monitor pulse signals at two distant locations. This can lead to two problems: inconvenience in operation and inaccurate measurement of the distance between the two positions. There are some studies indicating that the sensors can successfully monitor multichannel pulse waves through sensor array [41,42], which provides a good reason for subsequent work. As a new type of sensing principle first demonstrated by Prof. Zhong Lin Wang’s group in 2012, triboelectric nanogenerator is increasingly becoming a popular method for monitoring pulse wave because it is self-powered, cost-effective, and universally available [43]. Based on the broad selection of materials, the diversity of working modes, and the good sensing ability for weak signals, triboelectric pressure sensors also exhibit good application prospects in achieving low-cost and efficient preparation of pressure sensor arrays. However, there are some major challenges in developing a triboelectric-based flexible sensor with small area that can detect weak pulse wave, because it is difficult to ensure the effective contact–separation process of the sensor on a small and effective area. The design of a triboelectric-based flexible sensors array will be a good supplement to PWV monitoring based on flexible sensing technology.

Like flexible sensors based on other principles, to improve the output performance of the triboelectric-based sensor especially for sensors with small area, the modification of the tribo-pair surfaces by introducing microstructure is a very effective method. Previous research has shown that nanofibers are outstanding candidates for enhancing sensors’ output performance due to their many extraordinary properties such as high surface-to-volume ratio, reusability, and porous structure [44]. The nanofibers can be fabricated by electrospinning technology, which is an inexpensive, simple, and straightforward processing method that uses a spinnerette with electric field to force the polymer solution [45]. In addition to the above method of directly forming materials with microstructures by applying special technologies, this method is also an effective way to fabricate microstructures by copying the surface of objects with microstructure. Sandpaper with a rough surface is often used as the mold template to fabricate frosted microstructure, which is a convenient approach for forming a surface with rich morphology. Based on the electrospinning and turnover formwork, the electrification layers will be modified by nanofibers and frosted microstructure in a cost-effective, simple method, which is beneficial for forming a small sensors array with a good contact–separation area. This will provide a low-cost and efficient method for fabricating a triboelectric-based pressure sensors array.

Herein, we developed a 3 × 1 flexible pressure sensor array based on single-electrode triboelectric-based sensors for epidermal pulse wave monitoring at multiple locations of the radial artery. The single sensor with 6 × 6 mm^2^ is mainly composed of frosted microstructured Ecoflex film and TPU nanofibers. Rely on the electrospinning process, the micropatterned TPU nanofibers was fabricated on the FEP surface. The Ecoflex film with frosted microstructure are obtained by copying the microstructure of the sandpaper via turnover process. The flexible pressure sensor based on the above microstructure holds a high surface-to-volume ratio, exhibiting a sensitivity of 0.14 V/kPa, a response time of 22 ms, a low-frequency band ranging from 1 to 23 Hz, and stability up to 7000 cycles. The sensor characterized by the above features has been successfully applied in detecting body actions and manifold physiological signals including respiration, ballistocardiogram, and epidermal pulse waves. Importantly, the 3 × 1 flexible sensors array can simultaneously capture the pulse waves at different local positions of radial artery. The PTT and PWV characteristic parameters can be successfully extracted from the obtained pulse waveform. This not only reduces the requirements for location during measurement but also obtains more abundant physiological information, which is highly desirable in pulse-based cardiovascular system health monitoring.

## 2. Materials and Methods

### 2.1. Fabrication of the Frosted Microstructured Ecoflex Film

Prepare Ecoflex mixture according to the ratio of 1:1 (*w*/*w*). Fix the sandpaper (the mesh number is # 800) in the spin-coating machine. Pour Ecoflex mixture onto the sandpaper surface and spin it. The rotational speed is 1000 r/min, and the spin-coating time is 20 s. Store the prepared sample at room temperature for curing. The thickness of the formed frosted microstructured Ecoflex film is 0.16 mm.

### 2.2. Fabrication of the TPU Nanofibers by Electrospinning

Add 10 mL of Tetrahydrofuran (THF) solution, 4 mL of DiMethyl Formamide (DMF) solution, and 1.2 g of TPU particles to a beaker. Next, put a magnetic float in the beaker and seal the beaker with a plastic film. Then, fix the beaker on the magnetic stirrer. The magnetic stirrer operates for 30 min at 0 r/s and 50 °C and then for 2 h at 200 r/s and 50 °C. Fill the syringe with the obtained TPU mixed solution. Lay the glued FEP film on the metal plate. The positive and negative poles connected by the voltage source are connected with the needle and metal plate of the syringe, respectively. Adjust the output voltage of the voltage source to about 10 kV; the TPU nanofibers were obtained randomly on the surface of the FEP film. The thickness of the TPU nanofibers and the glue are about 0.02 and 0.01 mm, respectively.

### 2.3. Fabrication of the Sensor

First, connect the conducting wire on the Cu film. Then, glue the Cu film and the frosted microstructured Ecoflex together. Finally, place the frosted microstructure and TPU nanofibers opposite each other and use glue to bond them.

### 2.4. Testing System for Characterize the Output Performance

A function generator (SDG 2122X), a power amplifier (PA-1200), and a vibration shaker were used for generating a standard excitation signal. A high-precision force gauge (SBT630) was used to measure the external pressure. An electrometer (Keithley 6514) and a data acquisition card (NI BNC 2120) were utilized to capture output signals of the flexible sensor. The upper computer can be used to display and save data. Based on the testing system, the raw output waveforms of the sensor under different pressures can be obtained.

## 3. Results and Discussion

### 3.1. The Design of the Sensor with Small Area and Sensors Array

**Figure 1:**
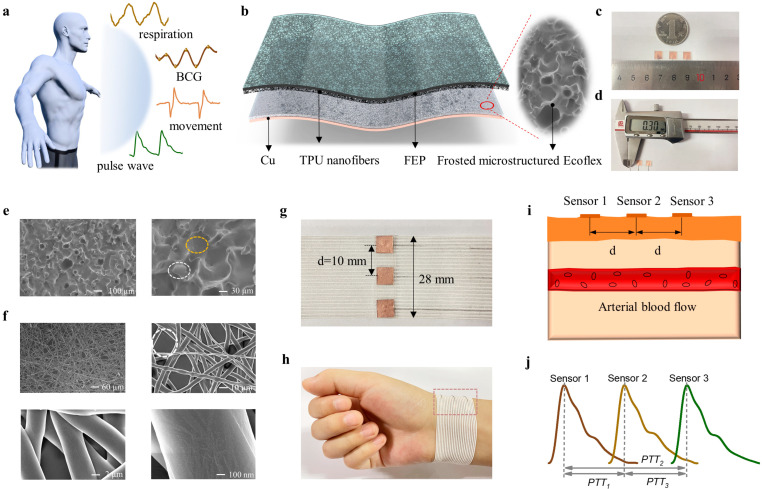
Construction of the sensor array. (**a**) Different kinds of human physiological signals include respiration, BCG, movement, and pulse wave. (**b**) Schematic structure diagram of the single sensor. (**c**,**d**) The dimension of sensor. (**e**) The SEM images of the frosted microstructured Ecoflex film, which consists of groove (marked by orange dotted box) and convex (marked by white dotted box). (**f**) The SEM images of the TPU nanofibers fabricated on the FEP surface. (**g**) The optical image of 3 × 1 flexible pressure sensor array and relative position relationship. (**h**) Photograph of a sensor array on the subject’s wrist to capture human epidermal pulse waves. (**i**) The sensor array placement configuration and human artery simplified diagram. (**j**) Schematic diagram of three pulse signals of radial artery position.

Various different bio-signals (e.g., motions, respiration, ballistocardiogram, and epidermal pulse waves) provided by the human body are usually used as indicators to judge individual health status, which can be successfully detected by the designed sensor in this work, as shown in Figure 1a. The designed sensor mainly holds a multilayer structure, as schematically shown in Figure 1b, as well as a frosted microstructured Ecoflex film as an electrification layer and a Cu layer as the back electrode for electrical connection with the external circuit. TPU nanofibers formed by electrospinning were fabricated on the FEP surface, serving as another electrification layer. Compared to a coin worth one yuan, the designed sensor has a significantly smaller size of 6 × 6 × 0.3 mm^3^ as illustrated in Figure 1c,d, which causes the sensor to be closely attached to the radial artery.

In the process of the sensor design, Ecoflex with surface morphology of frosted microstructure and TPU nanofibers prepared on the FEP surface play important roles in promoting sensors’ output performance. The scanning electron microscopy (SEM) images of the frosted microstructure are presented in Figure 1e. Notably, frosted microstructure is randomly distributed on the Ecoflex surface, forming the morphology of the combination of protrusion (marked by the white dashed box) and depression (marked by the orange dashed box). This greatly increases the contact–separation area between the two electrification layers. The detailed fabrication procedures of the frosted microstructured Ecoflex film are exhibited in Figure S1 and the Section 2. The thickness of the frosted microstructured Ecoflex film is 0.16 mm, as shown in Figure S2. The morphology of the TPU nanofibers was characterized by SEM images with different magnifications, as shown in Figure 1f. There is a clear fiber network in the random overlay process, and a diameter of the fiber ~4 μm is clearly presented. There are many pore structures exhibited among the different fibers, resulting in a high surface-to-volume ratio. The specific process of the electrospinning is illustrated in Figure S3 and the Section 2. The pore structure of nanofibers (marked by the dotted circle in Figure 1f) provides more possibilities for contact with the frosted microstructure. Moreover, nanofibers with layered structure can ensure that there are still pore structures that can contact the surface of the frosted microstructure under different pressures, which is conducive to the sensor responding to different dynamic pressures. A detailed fabrication process of the designed sensor is exhibited in Figure S4 and the Section 2.

To achieve reliable pulse wave measurements at the wrist, we fabricated a flexible pressure sensor array onto the wristband, as shown in Figure 1g. It includes a 3 × 1 array of the designed sensors with a size of 6 × 6 mm^2^. The length and width of the entire sensor array are 28 mm and 6 mm, respectively, and the center-to-center distance between two adjacent sensors is 10 mm. With its remarkable flexibility, the wristband with sensors array can be conveniently and easily worn on the individual’s wrist (Figure 1h). Based on the Velcro design, the array can be in close contact with the arterial position. The sensors array placement configuration and the human artery simplified diagram are shown in Figure 1i. Periodic external pressure results in a periodic contact–separation process between frosted microstructure and TPU nanofibers. Attributed to the electrostatic induction and charge transfer principle [46], electrons transfer between the reference electrode and the Cu electrode for charge balance, resulting in electrical output signals. Therefore, the pulse waves of the three local positions of the radial artery can be obtained simultaneously, which enables PTT measurement, as shown in Figure 1j.

### 3.2. The Characterization of Sensor Performance

The working principle of the sensor is illustrated in Figure S5. Assuming that the two materials are in close contact in the initial state, there is no output signals. A relative separation between the Ecoflex and the TPU will occur when the external pressure is released, causing a potential difference between the Cu and the reference ground. This causes electrons to flow from the reference ground to the Cu layer, producing an electrical output signal. The TPU will approach the Ecoflex when external pressure is applied to the sensor, which leads a reversed output signal. Owing to the electric potential difference, the electrons will flow from the Cu layer to the ground. There is no output signal when the positive charge on the electrode is fully balanced by the induced negative charges on the Ecoflex surface. When external pressure is applied to the sensor, the TPU will approach the Ecoflex, and the induced positive charge on the Cu electrode decreases, driving the electron flow from the ground to the Cu electrode until the two materials are completely in contact with each other again, leading to a reversed output signal.

**Figure 2:**
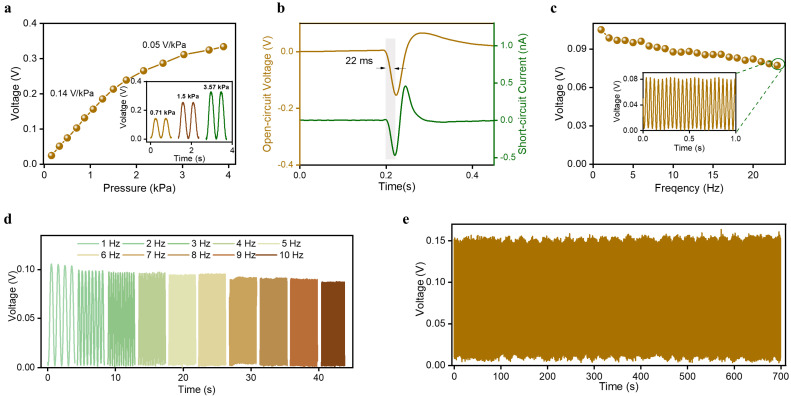
The output performance of the sensor. (**a**) The pressure sensitivity of the designed sensor. The inset shows the output waveforms of the sensor under three different pressures (0.71 kPa, 1.5 kPa, 3.57 kPa). (**b**) Response time characterization of the sensor. (**c**) The open-circuit voltage of the sensor under a pressure of 0.7 kPa at frequency of 1−23 Hz. The inset shows the output waveforms of the sensor (0.7 kPa, 23 Hz). (**d**) The output signals of the sensor under the same pressure with frequency ranging from 1 to 10 Hz. (**e**) The stability of the sensor under a pressure of 1.24 kPa with a frequency of 10 Hz.

To quantitatively characterize the output performance of the designed sensor, a measuring system containing a functional generator, a power amplifier, a vibration shaker, a force meter, an electrometer, a data acquisition card, and an upper computer was used, as shown in Figure S6 and the Section 2. During sensor’s output performance testing, the vibration of the shaker causes the sensor to come into contact with the force gauge fixed above the sensor, causing pressure to be applied to the sensor. The pressure applied on the sensor is precisely detected by the force meter, and the sensor’s output signals is finally saved by the upper computer using the electrometer and the data acquisition card. Pressure sensitivity is an important parameter for evaluating the sensor output performance. Based on the testing system, we first measured the output signals of the sensor under different pressures. As shown in Figure 2a, the amplitude of output waveform increases with the increasing pressure. It is observed that the output voltage in low pressure showed a pressure sensitivity (the ratio of the change in sensor output voltage to the corresponding pressure change) of 0.14 V/kPa, and lower-pressure sensitivity of 0.05 V/kPa was indicated with the further pressure increase. The inset shows the output waveforms of the sensor under three different pressures (0.71 kPa, 1.5 kPa, and 3.57 kPa). Under the stimulation of small pressure, the contact–separation area changes greatly with the increase of pressure, resulting in large sensitivity. The deformation of the sensor tends to saturate under high pressure, resulting in a decrease in the contact–separation area under the same pressure change, which reduces the sensitivity of the sensor.

Additionally, a further measurement was carried out to obtain the time taken to respond to the loaded pressure. Response time refers to the speed at which the sensor responds to external pressure signals, which can be calculated by the time difference and the time point at which the sensor generates an effective output signal under the excitation of a pulse signal and the first peak value of output signals [47]. The acquired short-circuit current waveform and open-circuit voltage waveform under the stimulation of the square wave signals with a duty ratio of 1% are shown in Figure 2b. The measurement results show a rapid current change when the external pressure was applied to the sensor surface, demonstrating a response time of about 22 ms. Considering that the main frequency components of human physiological signals are less than 10 Hz, we investigated the output voltage of the sensor under the same pressure (0.7 kPa) with the frequency ranging from 1 to 23 Hz. The measured frequency response is presented in Figure 2c, indicating that there is a decrease trend with the increased frequency and that there is no obvious distortion even under excitation signal of 23 Hz. Figure 2d shows the output voltage waveforms under the excitation of the pressure with frequency from 1 to 10 Hz. Therefore, the sensor is able to respond to human physiological signals. The output stability of the sensor is important for continuous monitoring in practical application scenarios. To characterize this, the measurement was conducted to record the output voltage of the sensor by loading/unloading at least 7000 cycles under a pressure of 1.24 kPa with a frequency of 10 Hz, as shown in Figure 2e. The first cycle and the last eight cycles during the measuring process are illustrated in Figure S7. The relative variation of the output voltage amplitude relative to the average amplitude during the acquisition time fluctuates between 94.5% and 104.7%, which indicated good stability of the sensor.

**Figure 3:**
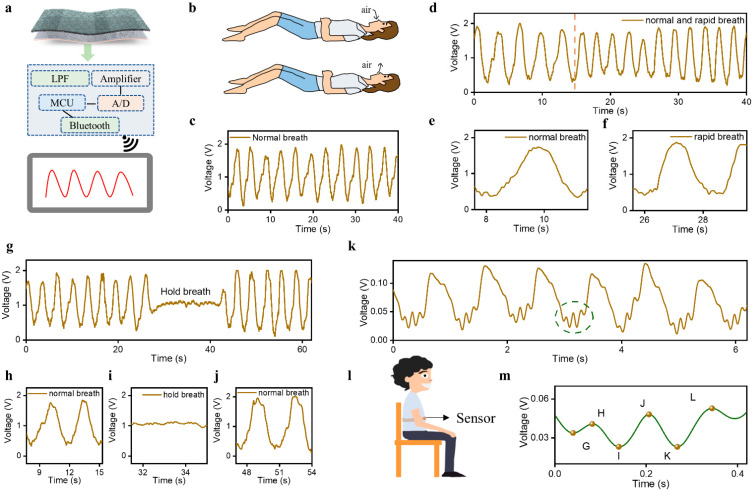
Multiple physiological signal monitoring. (**a**) Schematic diagram of pulse signal monitoring system composition. (**b**) The abdominal state of an individual during normal breathing. (**c**) Respiratory signals are measured by the sensor in normal breath. (**d**) The measured waveforms under two different breath conditions (normal and rapid breathing). (**e**,**f**) The respiratory signals of 3.9 s in different breath states (normal and rapid breathing). (**g**) Dynamic monitoring of respiratory signals under different states (**h**) normal breathing, (**i**) holding breathing, and (**j**) back to normal breathing. (**k**) BCG signals. (**l**) Schematic diagram of BCG test process. (**m**) A typical BCG waveform contains six prominent peaks: the presystolic (**g**), the systolic (**h**–**k**), and the diastolic (**l**).

Owing to the electrical and mechanical performance of the designed sensor, a wireless monitoring system was developed to achieve human physiological signal monitoring as shown in Figure 3a, which mainly includes a low-pass filter, an amplifier, and a wireless transmission module. The optical view of the hardware circuit system with a size of 2.7 × 2.6 × 0.5 cm^3^ is presented in Figure S8. In practical measurement, the designed sensor was utilized to acquire a raw physiological signal. The hardware circuit system filters and amplifies the raw signals and then transmits the data to the mobile terminal application through a Bluetooth module. Therefore, human physiological signal measurement can be realized in a simple and convenient way without any large-scale and complicated equipment. All physiological signals tested in this paper rely on this wireless monitoring system, which has an amplification of 10.

### 3.3. Physiological Signals Monitoring including Respiration, BCG, and Body Movement

Based on the sensing performance, the sensor was investigated for monitoring physiological signals including respiration, ballistocardiogram, and pulse waves. As a necessary physiological process for the exchange of gas between the body and the outside world, respiration plays a very important role in maintaining normal life activities. During breathing, dilation and contraction of the chest cavity are accompanied by inhaled and exhaled gas, as shown in Figure 3b, inducing the contact–separation process between tribopairs and then generating electrical signals that characterize respiration. First, we employed the sensor to capture the respiration signals of a 27-year-old female in supine posture with a period of 40 s, as shown in Figure 3c. The respiration waveforms with an amplitude of ~1.6 V were successfully obtained by the sensor. Although there are small fluctuations in the amplitude of the acquired signals, the signals are generally stable in a short time. The calculated respiratory rate was 17 breaths/min, which is consistent with the respiratory rate range of normal individuals. This verifies that the sensor has the ability to accurately represent the breathing process.

In addition, we utilized the sensor to record the respiratory signals with the breathing process of a 25-year-old female changing from normal to rapid, as shown in Figure 3d. It was observed that there are about 4 respiration cycles in 0–13 s and about 11 respiration cycles in 15–40 s. To clearly exhibit the respiration waveforms, the respiratory signals of 3.9 s in different states were selected for analysis, as shown in Figure 3e,f. Figure S9 presents the respiratory rate in normal and rapid breathing, which indicates that the rapid respiration rate (26 breaths/min) is 1.53 times higher than that in normal breathing (17 breaths/min). This shows that the sensor can capture respiration signals in different states. As a typical sleep-related disease in daily life, the incidence of obstructive sleep apnea-hypopnea syndrome (OSAHS) is very high. To prove whether the sensor is capable of monitoring OSAHS, we measured the respiration signals at three states (normal, hold breath, and normal), as shown in Figure 3g. The detailed waveforms under different states are illustrated in Figure 3h–j. It can be observed that there are tiny heartbeat signals and no respiration in the holding breath scenario. The real-time change trend of the respiratory rate is shown in Figure S10.

Except for respiration signals, the sensor also has the ability to acquire ballistocardiograph (BCG) signals with the help of the expansion belt. Figure 3k shows the test diagram where the sensor was fixed on the lower side of the right rib with a belt. The measured waveforms are shown in Figure 3l, which consists of two kinds of signals. One is the heartbeat, which holds an amplitude of about 0.1 V. The other is the BCG signal with a small amplitude of 0.03 V. The BCG signal of one cycle in Figure 3k is shown in Figure 3m, which presents six obvious peaks including the presystolic (G), the systolic (H, I, J, K), and the diastolic (L). This suggests that the designed sensor holds promising potential in cardiovascular system monitoring.

### 3.4. Pressure Sensors Array for Pulse Wave Monitoring

**Figure 4:**
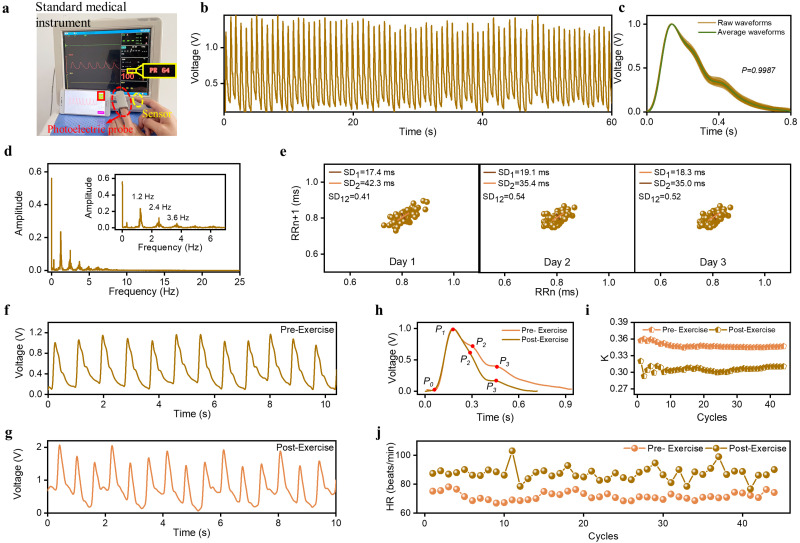
Human pulse wave monitoring. (**a**) Pulse wave monitoring by standard medical instrument and sensors simultaneously. (**b**) Continuous pulse wave monitoring for 1 min. (**c**) Consistency of pulse signals measured within 1 min. (**d**) The frequency spectrum of a 27-year-old woman’s radial artery pulse signals. (**e**) Pulse signal monitoring for continuous 3-day cycle. (**f**) The pulse wave monitoring before exercise. (**g**) The pulse wave monitoring after exercise. (**h**) The enlarged view of one cycle pulse signal measured before and after exercise. (**i**) Change of the K value before and after exercise. (**j**) Change of the HR before and after exercise.

As one of the most representative human physiological signals, pulse waves cover rich information about the cardiovascular system and have been widely used in clinical examinations for disease diagnosis, especially in traditional Chinese medicine. Noninvasive and convenient pulse signal monitoring is beneficial to the early diagnosis and prevention of cardiovascular diseases. Based on frosted microstructure and nanofiber structure, the designed sensor shows great ability to realize pulse wave monitoring. To verify the accuracy of the sensor monitoring pulse signals, we used a commercial standard medical instrument (MHM-6000B) and the sensor with small area to acquire the same person’s fingertip pulse waves simultaneously, as shown in Figure 4a. In the actual testing process, the photoelectric probe and the sensor are worn on the left index finger and the right index finger of the individual, respectively. The measured result shows that the pulse waves measured by these two devices have good similarity in waveform shape and consistent heart rate. This proves that the sensor has the ability to accurately monitor pulse waves. In addition, we attached sensor to the individual’s (27-year-old female) wrist, and the measured waveforms with a period of 60 s are shown in Figure 4b. Although they fluctuated in amplitude, the signals are stable on the whole. Figure 4c presents the superposition diagram of the normalized waveforms of different cycles, which exhibit three obvious peaks: advancing wave peak (P_1_), reflected wave peak (P_2_), and dicrotic wave peak (P_3_). The average Pearson correlation coefficient among different cycles is as high as 0.9987, suggesting that the developed sensor can achieve pulse wave monitoring stably. The spectrum information of pulse signal is also of great significance for the assessment of cardiovascular system status. Figure 4d shows the power spectra of the obtained pulse waves, which contains three main components: 1.2, 2.4, and 3.6 Hz. It can be observed that the amplitude of harmonic components exhibited a decreasing trend with increasing frequency. The fundamental frequency component in the spectrum represents the frequency of the heartbeat, which is consistent with the average heart rate obtained from the pulse waveforms in the time domain (72 beats/min). To evaluate the stability of the sensor in different pulse wave measurements, we utilized Poincare plots to intuitively describe the dispersion of pulse intervals. The Poincare plot is mapped by scatter points ((R1, R2), (R2, R3) … (Rn−1, Rn), (RRn, RRn+1)), where RRn is approximately considered as the pulse interval between the nth and next successive pulse waves [37]. The SD_1_ and SD_2_ can reveal variability of pulse interval in the short term and the long term. For a continuous 3-day measurement, the dispersion of the pulse wave interval of a healthy subject is small, with SD_1_ ranging from 17.4 to 19.1 ms and with SD_2_ ranging from 17.4 to 19.1 ms, as shown in Figure 4e. SD_12_ in a 3-day measurement were also obtained, which are 0.41, 0.54, and 0.52. These data all present a healthy heart state.

The exercise process has a certain impact on the pulse waves. To characterize this, we acquired the pulse waves at wrist pre-exercise and post-exercise, as shown in Figure 4f,g. Compared with signals pre-exercise, the measured pulse waves post-exercise has higher amplitude and is not stable, which is due to the more severe respiratory signal superimposed on the measured pulse signal. Furthermore, there are obvious difference in shape between pulse wave pre-exercise and post-exercise. As illustrated in Figure 4h, the waveform obtained after exercise has a smoother falling slope and only holds one obvious peak P_1_. As an important indicator of physiological state proposed by Prof. Luo, the *K* value can reflect physiological and pathological states, which can be obtained by the following formula [38]:

$$K=\frac{P_{m}-P_{0}}{P_{1}-P_{0}}$$

where $P_{m}=\frac{1}{T}\int_{0}^{T}p(t)dt$ and T is the cardiac cycle. As shown in Figure 4i,j, exercise leads to a lower K and a higher HR. Exercise can accelerate blood circulation and enhance cardiac blood-ejection ability, which requires the heart to pump blood quickly to meet the needs of exercise, leading to an increase in HR and a decrease in K value. This indicates that the sensor designed in this paper is helpful for objectively evaluating the impact of exercise on physiological activities. Furthermore, the pulse waves at the wrist of different individuals can also be measured (Figure S11). In addition, we measured the pulse waves at fingertip and ankle (Figure S12), which indicates that the sensor can be applied to different arterial position to adapt to different application scenarios.

### 3.5. Pressure Sensors Array for Pulse Wave Monitoring

**Figure 5:**
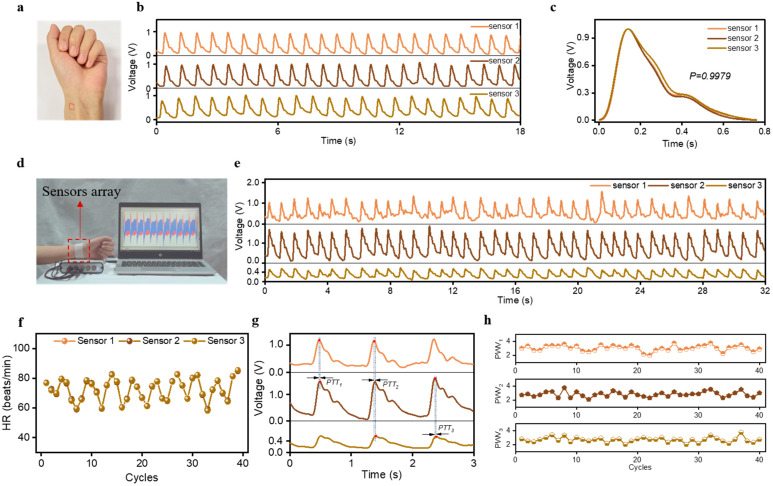
A flexible sensor array for monitoring pulse signals at multiple local positions of the wrist. (**a**) Pulse wave monitoring position indicated by marker. (**b**) The measured waveforms by the three sensors in sensor array at the same position. (**c**) The consistency of the signals measured by the three sensors. (**d**) The optical image of pulse wave monitoring by sensor array. (**e**) The measured pulse wave signals by sensor array. (**f**) Real-time HR value extracted from pulse signals obtained at three local locations. (**g**) The enlarged view of the pulse wave at three local locations and schematic of different PTTs. (**h**) The change trend of PWV value in 40 cycles.

Based on the remarkable ability of the sensor to monitor pulse signals, we formed a 3 × 1 flexible sensor array. It is easy to located the center of the sensor on the artery for pulse wave signal acquisition because the sensor size is small. In order to prove the consistency of the pulse signals monitored by these three sensors, we used the sensors to measure the pulse signals at the same position. Before the measurement, we used a marker to indicate a fixed test position at the subject’s wrist to ensure consistency of the test position, as shown in Figure 5a. In a practical measuring process, the subject keeps the posture unchanged. The real-time radial pulse waves of a 27-year-old individual were measured by three sensors with a period of 18 s, as shown in Figure 5b. It can be observed that the three sensors can monitor pulse signals stably, and these signals show good similarity in amplitude and shape. The consistency of the signals measured by these three sensors are shown in Figure S11. Figure 5c shows the normalized average waveforms of the captured signals in Figure 5b. Compared with the signals from Sensor 1 and Sensor 2, the signal from Sensor 3 shows a higher amplitude in P_1_ and P_2_. This phenomenon may be due to the difference of pulse signals at different times. However, the time when the characteristic points of the three signals appear has good consistency, and the Pearson correlation coefficient between these three average waveforms is as high as 0.9979, proving that the three flexible sensors in array have the ability to consistently monitor pulse signals.

The sensors array was located at the radial arteries with the help of the wristband for capturing the arteries’ pulsatile activity, as shown in Figure 5d. Sensor 2 is placed at the position with the largest pulsation intensity of the radial artery. The measured pulse waveforms in 32 s are shown in Figure 5e. The HR obtained from the three pulse waves have very good consistency in the same cycle, as shown in Figure 5f. This is because the two pulse waves whose peaks are close in time come from the same cardiac cycle; that is, the HR of the two pulse signals obtained at the same time is the heartbeat frequency at that time, which is in line with the individual physiological properties. The enlarged view shows the pulse waveforms in three cycles, exhibiting distinguishable feature points (Figure 5g). The signals from Sensor 2 shows the maximum amplitude and the best stability, which is due to the pulse signal sensed by Sensor 2 being relatively strongest. There is a slight difference in amplitude of feature points among pulse waves measured by the sensors array, which is caused by the pulse waves of different parts in the corresponding tissue organizational structure and transmission path. As an effective parameter for evaluating cardiovascular health and degree of the arterial stiffness, the PTT is also obtained by calculating the time difference between pulse signal peaks at different positions in the same cardiac cycle, as shown in Figure S12. Since the center distance between the two adjacent sensors is 10 mm, the average values of 3.03, 2.82, and 2.69 m/s for PWV_1_, PWV_2_, and PWV_3_, respectively, were obtained, as shown in Figure 5h. These three kinds of PWV show the same change trend, which is consistent with the physiological activities. These results are indicative of the ability of the multiple measurement of PWV using our proposed 3 × 1 flexible sensors array.

## 4. Conclusions

In this work, a triboelectric-based 3 × 1 flexible pressure sensor array was developed for pulse wave signal acquisition. The single sensor has a small size of 6 × 6 mm^2^ and mainly consists of frosted microstructure and TPU nanofibers fabricated by the electrospinning process. Featured with a sensitivity of 0.14 V/kPa, a response time of 22 ms, low-frequency bands ranging from 1 to 23 Hz, and stability up to 7000 cycles, the single sensor exhibits a remarkable capacity for human physiological signals acquisition, including respiration, BCG, and epidermal pulse waves. Most importantly, the 3 × 1 flexible sensors array can be applied to monitor pulse waves at multiple positions of the radial artery, which provides a convenient platform for acquiring rich physiological information (including PTT and PWV). This will reduce the requirement for measurement location, which is highly desirable in pulse-based cardiovascular monitoring.

## Data Availability

No new data were created or analyzed in this study. Data sharing is not applicable to this article.

## Supplementary Materials

The following supporting information can be downloaded at https://www.mdpi.com/article/10.3390/s23073717/s1, Figure S1: The detailed fabrication procedures of the frosted microstructured Ecoflex film; Figure S2: The thickness test photo of the Ecoflex film. Scale bar, 1 cm; Figure S3: The detailed fabrication procedures of the TPU nanofibers; Figure S4: The detailed fabrication process of the designed sensor; Figure S5: The working principle of the designed sensor; Figure S6: A measuring system containing a function generator, power amplifier, vibration shaker, force gauge, electrometer, data acquisition card, and upper computer; Figure S7: The stability of the sensor. Figure S8: The optical view of the hardware circuit system with size of 2.7 × 2.6 × 0.5 cm^3^ and the connection of the sensor and circuit; Figure S9: The corresponding respiratory rate under two different breath conditions (normal, rapid breathing); Figure S10: The corresponding respiratory rate under three different states (normal breathing, holding breathing, and back to normal breathing); Figure S11: The pulses wave at wrist of different individuals; Figure S12: The measured pulse waves at fingertip and ankle; Figure S13: The consistency of the measured pulse waves by single sensor; Figure S14: The change trend of PTT obtained by calculating the time difference between pulse signal peaks at different positions in the same cardiac cycle; Table S1: Comparison of pulse pressure sensor performance.
