# Supplementary material for: Flexible Sensors Array Based on Frosted Microstructured Ecoflex Film and TPU Nanofibers for Epidermal Pulse Wave Monitoring
Source: Sensors (Basel). 2023 Apr 3;23(7):3717. doi: 10.3390/s23073717 (PMC10099249; doi:10.3390/s23073717)
Supplement: Supplementary file 1 [file sensors-23-03717-s001.zip › sensors-2316667-supplementary.pdf]

Supplementary Materials

Flexible sensors array based on frosted microstructured Ecoflex film and TPU nanofibers for epidermal pulse waves monitoring

Xue Wang[1,2], Zhiping Feng[1,2], Gaoqiang Zhang[1,2], Luna Wang[1,2], Liang Chen[1,2], Jin Yang[1,2,*], Zhonglin Wang[3,*]

[1]State key laboratory of coal mine disaster dynamics and control, Chongqing University, Chongqing 400044, P. R. China.

[2]Key Laboratory of Optoelectronic Technology and Systems Ministry of Education, Department of Optoelectronic Engineering, Chongqing University, Chongqing 400044, China

[3]Beijing Institute of Nanoenergy and Nanosystems, Chinese Academy of Sciences, Beijing 100083, P. R. China.

*Authors to whom correspondence should be addressed. Electronic mail:

Email: yangjin@cqu.edu.cn (J.Y.),

Email: zhong.wang@mse.gatech.edu;

Supporting Figures and Captions

Table S1 Comparison of pulse pressure sensor performance

Principle	Structure	Sensitivity	Response time	Area	Array	Reference
piezoresistivity	nanofibers	-	-	8 × 5 cm^2	No	15
	bumps	10.53 kPa^{-1}	60 ms	2 × 2 cm^2	No	16
	microstructured fibroin	-	-	8 × 6 cm^2	No	17
	fabric	0.585 kPa^{-1}	4 ms	-	No	18
	urchin-like microstructure	680 kPa^{-1}	10 ms	>1 cm^2	No	19
	micropyramid	10.32 kPa^{-1}	-	0.9 × 0.9 cm^2	No	20
	hierarchically microstructure	53 kPa^{-1}	38 ms	~1 × 1.5 cm^2	No	21
	crack	>10^7 ΩkPa^{-1}	-	~1 × 0.5 cm^2	No	22
capacitance	paper	0.23 kPa^{-1}	41 ms	-	Yes	23
	microhairy	0.58 kPa^{-1}	30 ms	-	No	24
	porous Pyramid	44.5 kPa^{-1}	-		Yes	25
	pyramid	4.5 kPa^{-1}	50	14 × 4 mm^2	Yes	26
	Iontronic	13.5 kPa^{-1}	30 ms	10 × 10 mm^2	No	27
	pyramid	41 kPa^{-1}	20 ms	8 × 8 cm^2	No	28
	nanofibrous	5.5 kPa^{-1}	70.4 ms	5 × 5 cm^2	Yes	29
	airgap	1.277 kPa^{-1}	100 ms	20 × 20 mm^2	No	30
piezoelectricity	PZT	0.018 kPa^{-1}	60 ms	-	No	31
	PZT	1.36 uA Pa^{-1}	0.1 ms	-	Yes	32
	sandwich	32.6 nA/kPa^{-1}	18.6 ms	6 × 4 cm^2	Yes	33
	microdome	47.7 kPa^{-1}	20 ms	~1× 1.5 cm^2	No	34
triboelectric	3D dielectric geometry	0.33 $VkPa^{-1}$	-	15 × 15 mm^2	No	35
	curved surface	46.9 V/N	72 ms	2 × 2 cm^2	No	36
	hierarchical elastomer	7.989 $VkPa^{-1}$	40 ms	1 × 1 cm^2	Yes	37
	weaving structure	45.7mVPa−1	5	1 × 1 cm^2	No	38
	nanostructure	-	-	2 × 1 cm^2	Yes	39
	multilayer structure	10.29 nA/kPa		~2 × 1 cm^2	No	40
This work	frosted microstructure nanofibers	0.14 V/kPa	22 ms	0.6 × 0.6 cm^2	Yes	-

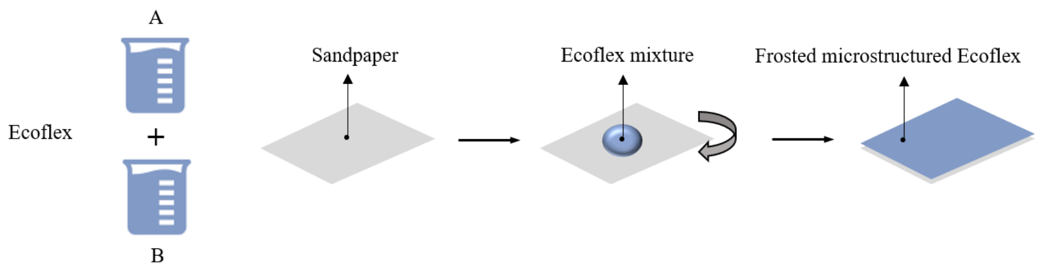

Figure S1. The detailed fabrication procedures of the frosted microstructured Ecoflex film.

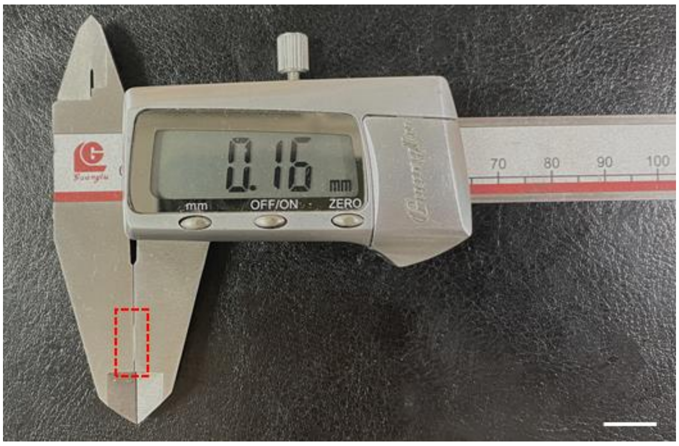

Figure S2. The thickness test photo of the Ecoflex film. Scale bar, 1 cm.

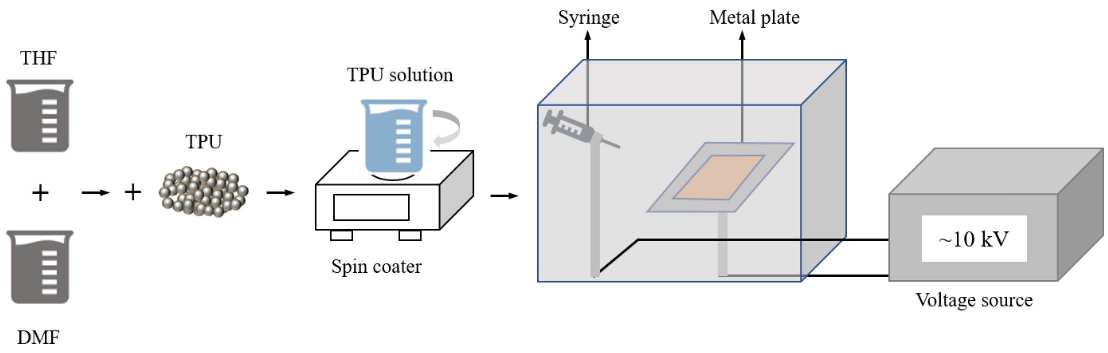

Figure S3. The detailed fabrication procedures of the TPU nanofibers.

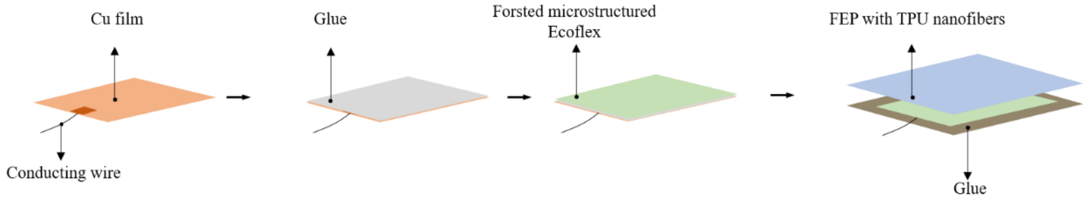

Figure S4. The detailed fabrication process of the designed sensor.

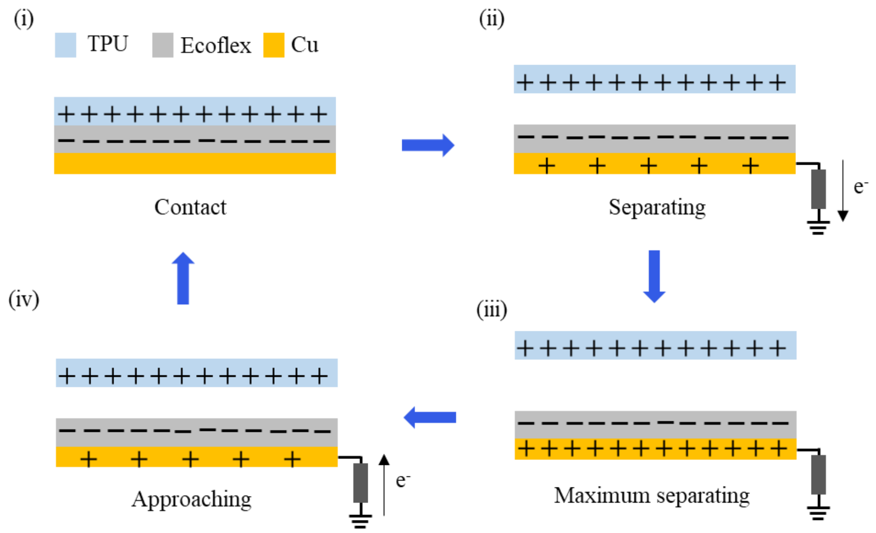

Figure S5. The working principle of the designed sensor.

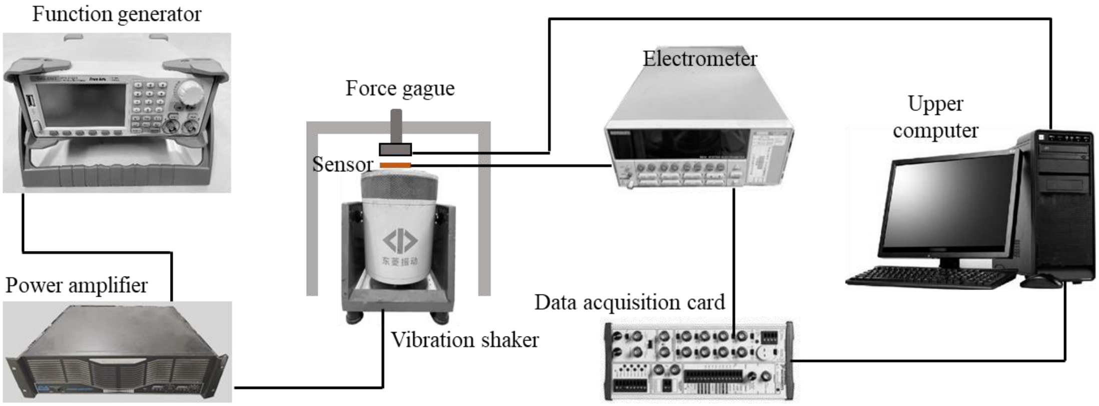

Figure S6. A measuring system containing a function generator, power amplifier, vibration shaker, force gauge, electrometer, data acquisition card, and upper computer.

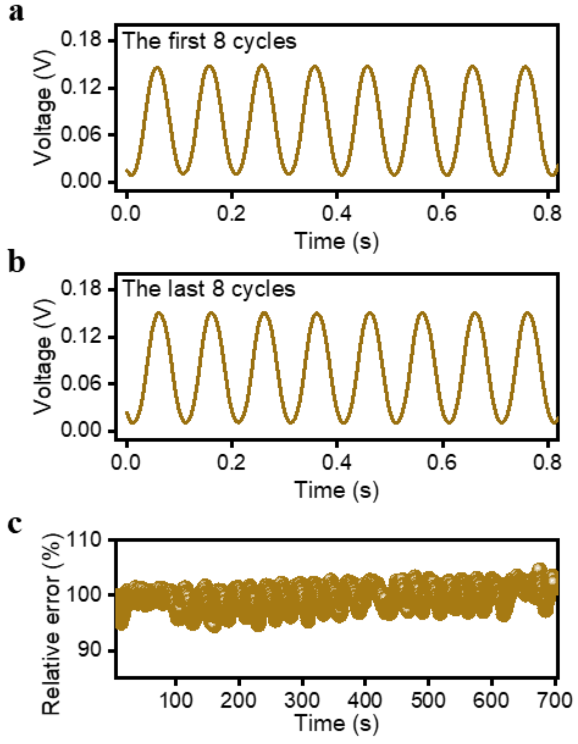

Figure S7. The stability of the sensor. (a) The first and (b) last 8 cycles in 7000 cycles consecutive measurement. (c) The percentage variation of the output voltage amplitude during the acquisition time.

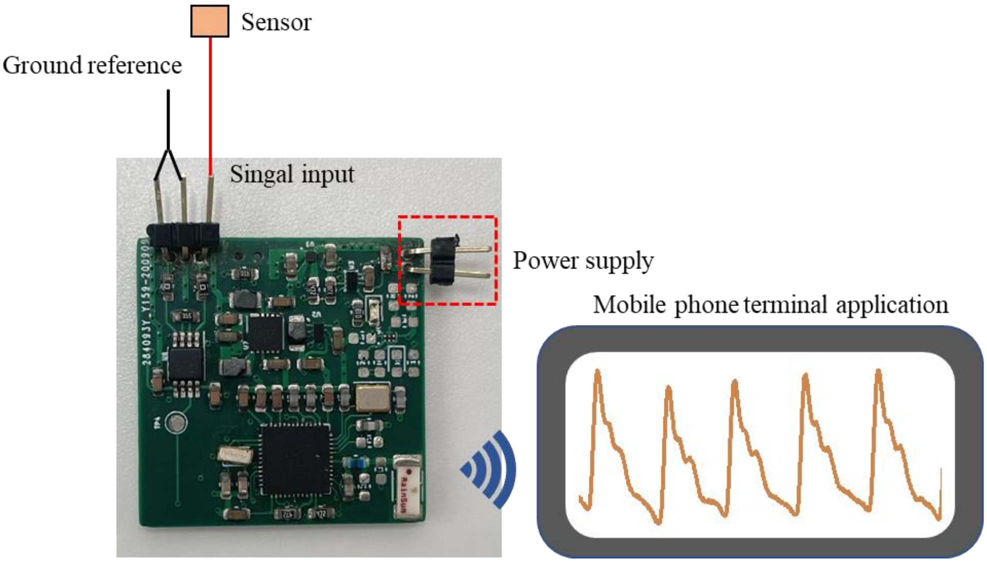

Figure S8. The optical view of the hardware circuit system with size of 2.7 × 2.6 × 0.5 cm^3 and the connection of the sensor and circuit.

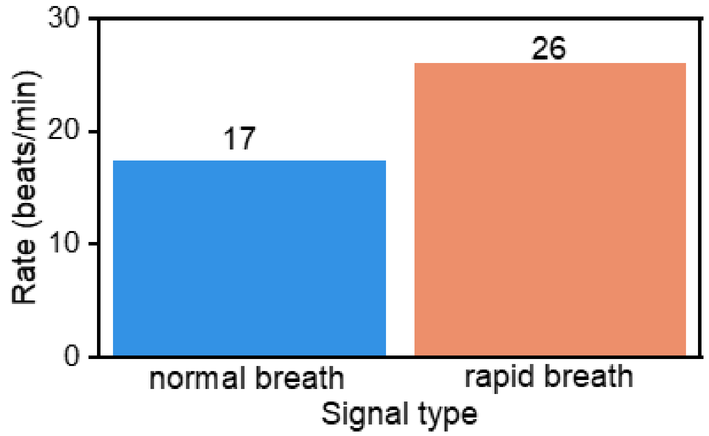

Figure S9. The corresponding respiratory rate under two different breath conditions (normal, rapid breathing).

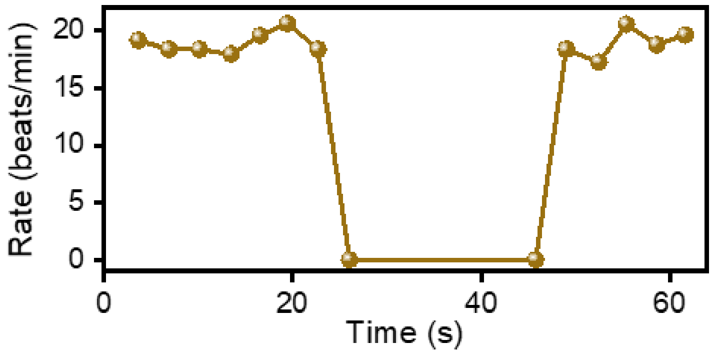

Figure S10. The corresponding respiratory rate under three different states (normal breathing, holding breathing, and back to normal breathing).

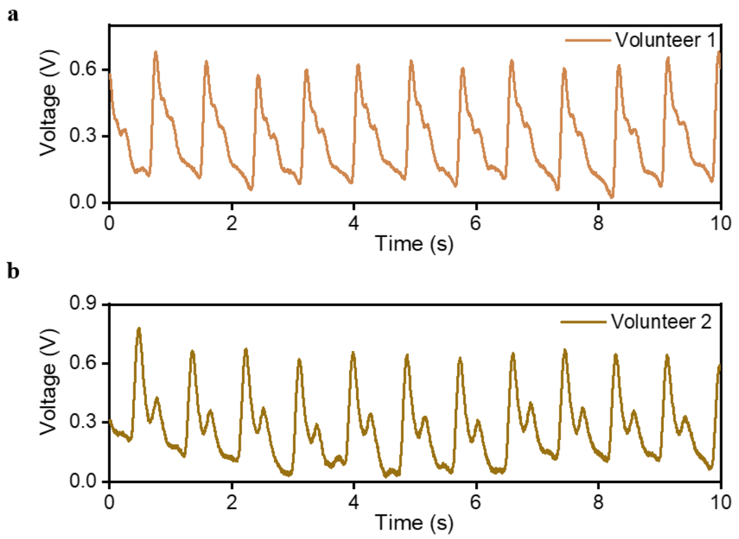

Figure S11. The pulses wave at wrist of different individuals.

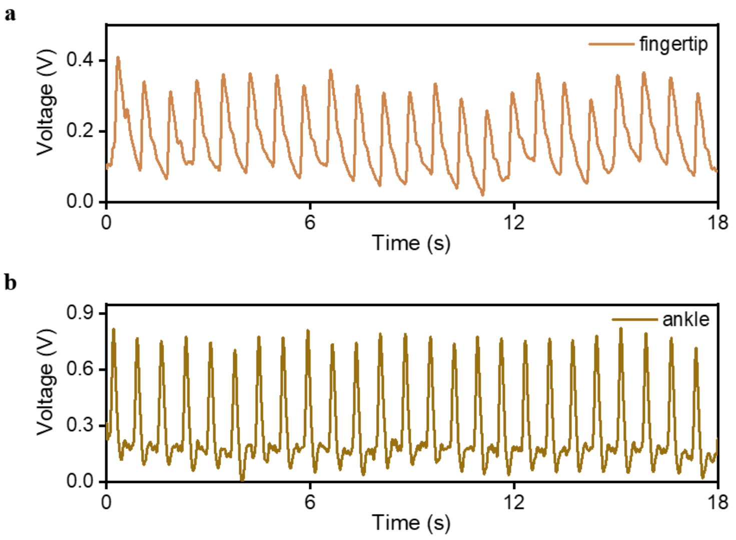

Figure S12. The measured pulse waves at fingertip and ankle.

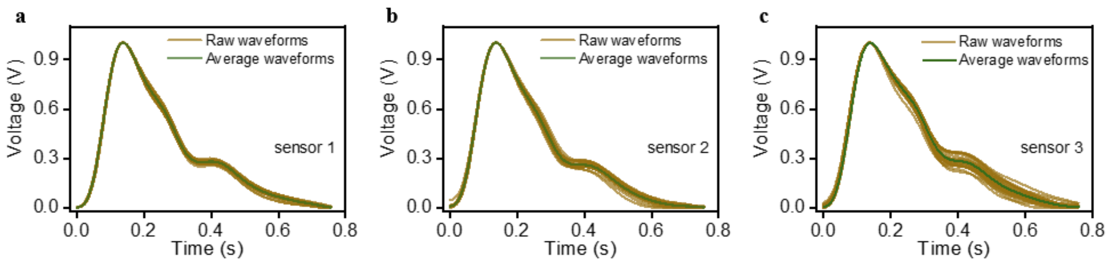

Figure S13. The consistency of the measured pulse waves by single sensor. (a) The consistency of the pulse signals measured by sensor 1 in the same measurement process. (b) The consistency of the pulse signals measured by sensor 2 in the same measurement process. (c) The consistency of the pulse signals measured by sensor 3 in the same measurement process.

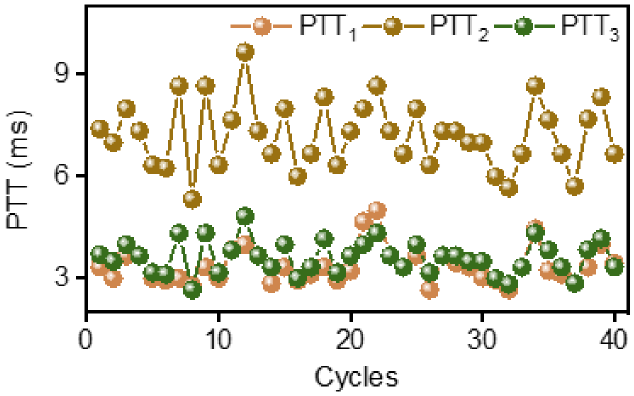

Figure S14. The change trend of PTT obtained by calculating the time difference between pulse signal peaks at different positions in the same cardiac cycle.
